# Social Needs and Type 2 Diabetes in Latinos: An Integrative Review

**DOI:** 10.1007/s40615-025-02448-z

**Published:** 2025-05-02

**Authors:** Aliria M. Rascón, Eyitayo O. Owolabi, Morgan E. Braxton, Niko Verdecias-Pellum, Gabriel Q. Shaibi

**Affiliations:** 1 Edson College of Nursing and Health Innovation, Arizona State University, Health North Suite 300, 550 N 3rd Street, Phoenix, AZ 85004 USA; 2 College of Health Solutions, Arizona State University, Phoenix, USA

**Keywords:** Diabetes, Hispanic/Latino, Social needs, Social determinants of health

## Abstract

Type 2 diabetes (T2D) disparities in Latinos in the United States continue to rise despite ongoing efforts to advance health equity. Major drivers of T2D disparities are shaped by the social determinants of health which create unmet social needs such as unstable housing, unreliable transportation, food insecurity, financial need, and insufficient childcare. Latino communities are disproportionately affected by many social determinants of health and thus report disproportionately greater social needs than their Non-Hispanic White counterparts. As T2D prevalence, incidence, and associated complications in Latinos outpace those of Non-Latino Whites, an understanding of the potential influence of social needs on T2D prevention and management in this population is warranted. This integrative review describes the role of social needs in T2D prevention and management among Latinos. This review informs how certain social needs are associated with increased risk for T2D, prediabetes, and poor T2D self-management. Specific social needs interventions had mixed results in affecting T2D outcomes and social needs. There is a lack of research evaluating interventions with comprehensive social needs screen and referral for Latinos with or at risk for T2D across the lifespan.

## Introduction

Type 2 diabetes (T2D) disparities in Latinos in the United States (US) persist despite ongoing efforts to advance health equity. The age-adjusted prevalence of T2D in adult Latinos is 12.5% compared to 7.5% of Non-Hispanic Whites [1]. Social determinants of health (SDoH) such as socioeconomic status are the root causes of T2D disparities [2]. SDoH are the social, political, and economic conditions and environments in which people “are born, live, learn, work, play, worship, and age” that impact health [3]. Specific adverse SDoH that disproportionately affect Latinos (e.g., lower levels of education, lower income, and residing in neighborhoods where outdoor recreation is perceived as less safe) are associated with poor T2D outcomes [2]. At the individual level, SDOH create “social risks” and “social needs.” While these terms are closely related, and often used interchangeably, they carry distinct differences. Social risks describe specific adverse conditions or exposures experienced by an individual that have been associated with poor health. Someone may not have a vehicle or live near a grocery store which increases the possibility of poor health outcomes; these are social risks. When an individual endorses specific needs as priorities in their life at that time such as needing transportation or help with food insecurity, these are social needs. A person may have many social risks but only endorse certain social needs [4]. While categories of what constitutes social needs vary throughout the literature, commonly referenced social needs include stable or safe housing, transportation, childcare, utility payment assistance, safety, employment, and food security [5]. Unmet social needs have been linked with unhealthy behaviors, less consumption of fruits and vegetables, and less exercise [6] and poor health outcomes such as depressive symptoms, diabetes distress, reduced healthcare access, greater diabetes-related hospitalization rates, and mortality [7–9]. Healthcare systems have increasingly incorporated screening of social needs as standard practice in order to be able to intervene and mitigate risk for health complications [10].

Sociocultural context shapes a person’s endorsement of social needs as well as interaction with associated resources. Cultural norms, structural discrimination, and historical factors influence sociocultural context—all of which are influenced by ethnicity. As the largest minority group in the US, Latino communities face greater social needs than their Non-Hispanic White counterparts [11] due to the disproportionate impact of various SDoH [12, 13]. As T2D prevalence, incidence, and associated complications in Latinos outpace those of Non-Latino Whites [14], an understanding of the potential influence of social needs on T2D prevention and management in this population is warranted. A focus on social needs rather than social risks emphasizes the person-centered approach necessary to understand individual experiences and preferences among Latinos at risk for or diagnosed with T2D. Therefore, this integrative review seeks to synthesize the current state of the evidence on how social needs influence T2D prevention and management in Latino populations.

## Methods

### Search Strategy

This integrative review was conducted between January and March 2024 utilizing Covidence data management software [15]. A search strategy was developed in consultation with a health sciences librarian and conducted in the following databases: PubMed, CINAHL, Sociological Abstracts, SocIndex, and Scopus (Table 1).

**Table 1:** Search strategy

Concept	Search terms
Population: Latinos in the US	Title/Abstract: Latin* OR Hispan* OR Chican* OR Mexic* OR Mexican American OR Colombia* OR Dominican* OR El Salvador* OR Cuba* OR Puerto Ric* OR Hondura* OR Venezuela* OR Bolivia* OR Peru* OR Ecuador* OR Costa Rica* OR Paraguay* OR Uruguay* OR Argentina* OR Chile* OR Guatemal* OR Nicaragua* OR Panama*
AND Exposure: with an identified social need (housing, transportation, childcare, employment, safety, legal assistance, food, utilities, income)	Title/Abstract: “Social need” OR “social needs” OR “basic need” OR “basic needs” OR “social risk” OR “social risks” OR “Social determinants of health” OR SDoH OR housing OR homeless OR unhoused OR transportation OR vehicle OR car OR childcare OR “child care” OR daycare OR “day care” OR Employ* OR job OR occupation OR work Legal OR law OR Safety OR abuse OR “intimate partner violence” OR IPV OR crime OR Utilities OR electricity OR “utilities insecurity” OR “Food insecurity” OR “food desert” OR “low income” OR poverty OR “financial hardship” OR “financial strain” OR Social Determinants of Health [MeSH]
AND Outcome: type 2 diabetes prevention or management	Title/Abstract: Diabetes OR diabetic OR “type 2 diabetes” OR T2D OR “type II diabetes” OR T2DM OR NIDDM OR “noninsulin dependent” OR “non insulin dependent” OR “non-insulin dependent” OR “adult onset diabetes” OR prediabetes OR prediabetic OR “pre-diabetes” OR “pre diabetes” OR Diabetes Mellitus Type 2 [MeSH]

### Screening

Articles from all searches were uploaded to Covidence Data Management Software and duplicates were removed. Three health equity scholars (AMR, EOO, MEB) analyzed the remaining articles by title, abstract, and full text using the following inclusion criteria: (1) qualitative and quantitative research evaluating one or more social need(s), which included housing, transportation, childcare, employment, safety, legal, food security, utilities, financial, and social support; (2) sample is either all Latino or data is disaggregated so that results for participants of Latino ethnicity can be evaluated separately; (3) specific social need(s) are either described/measured or interventions address social need(s); (4) outcome measures include glycemic indicators of T2D, T2D diagnosis or prediabetes, behaviors specific to T2D prevention, or self-management behaviors (healthy coping, healthy eating, being active, taking medication, monitoring, reducing risk, problem solving) [16].

Because the COVID- 19 pandemic influenced many social needs [17], articles for this review were included if published between 2017 and 2024 to represent approximately 3 years of research prior to and after declaration of the COVID- 19 pandemic. Considering that social needs are shaped by federal laws, public policy, and other societal factors, only research conducted in the US was included. Textbooks, dissertations, doctoral projects, seminars, conference abstracts, editorials, review articles, and case studies were excluded. Articles were also excluded if they were not available in English, full-text unavailable, or focused on type 1 diabetes, maturity onset diabetes of the young (MODY), latent autoimmune diabetes of adults (LADA), or gestational diabetes (GDM).

Throughout the screening process, reviewers engaged in roundtable discussions on the distinction between SDOH, social risks, and social needs and how these were operationalized within articles. Thus, while some articles provided contextual community-level information about SDOH and social risks, articles were excluded if they did not gather data at the individual level (e.g., subjective data), and thus did not collect data related to endorsing social needs.

### Data Extraction and Analysis

Two independent reviewers extracted data from each article, which was then reviewed and consolidated by a third reviewer. Level of evidence was determined using the Melnyk Hierarchy of Evidence Model [18] and quality of evidence was evaluated by two independent reviewers using the Mixed Methods Appraisal Tool (MMAT) version 18 [19]. Each article was also evaluated to determine its stage within the translational science spectrum, which reflects progress in bringing interventions to the people who need them in real-world settings and applications [20]. Data extracted from articles included setting, sample, design, data collection, which social need(s) were evaluated, if a social needs-related intervention was included, T2D-related outcome, whether the sample was only Latino or included multiple ethnicities, and whether Latin country of origin was described. Although terms such as “Hispanic,” “Latino,” “Latinx,” and “Latine” are often used interchangeably, these terms carry distinct definitions. Thus, article findings are reported using terminology described by the authors of each study. Data were analyzed using descriptive statistics and a narrative synthesis of articles was conducted using content analysis.

## Results

**Fig. 1:**
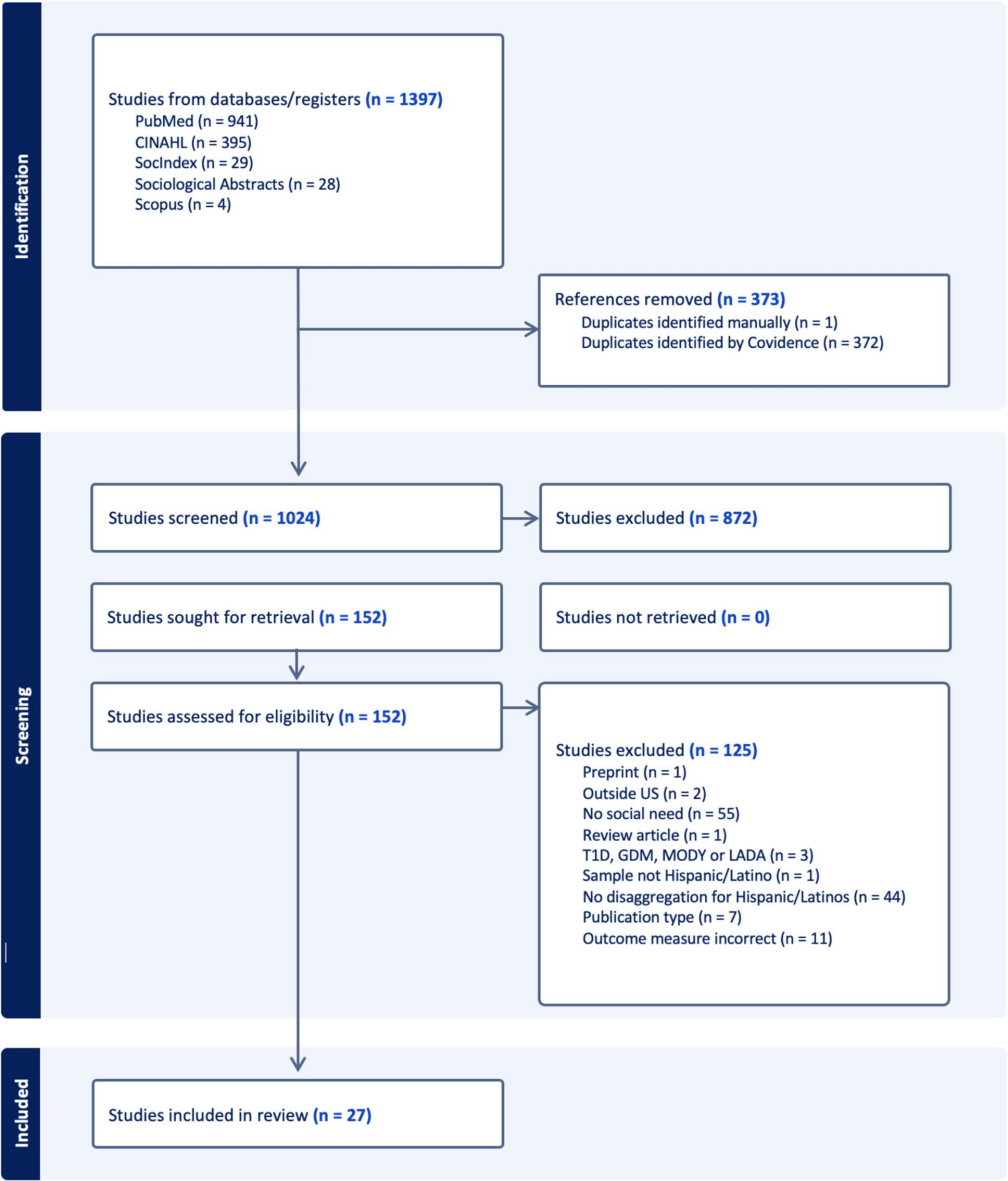
PRISMA diagram. Note: Generated by Covidence software

**Fig. 2:**
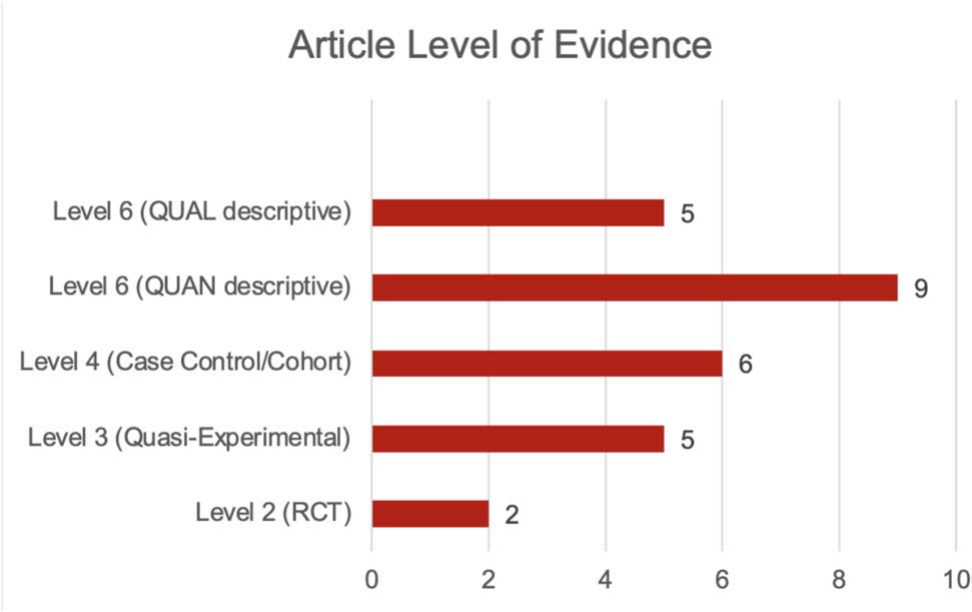
Level of evidence. Note: Using Melnyk Hierarchy of Evidence Model. No Level 1 or Level 5 studies (systematic reviews or meta-syntheses) included

Search results produced 1397 articles. After 373 duplicates were removed, 1024 articles were screened by title and abstract; of those, 872 were excluded. After full text review of the remaining 151 articles, 122 were excluded. The primary reasons for exclusion were not measuring a social need (*n* = 55) or not disaggregating data for evaluation of Latino participant outcomes (*n* = 44) (see Fig. 1). A total of 27 articles met inclusion criteria after completing all stages of screening. The majority of articles (*n* = 14) were descriptive in nature, either qualitative (*n* = 5) or quantitative (*n* = 9) with only two randomized controlled trials (see Fig. 2). Although the majority of articles were published after declaration of the COVID- 19 pandemic in 2020 (*n* = 16), only two studies reported on data collected after the COVID- 19 pandemic and neither compared pre—post pandemic differences [21, 22]. All articles were classified in the “Clinical Research” stage of the Translational Research Spectrum [20]. Critical appraisal of the evidence using the MMAT version 18 criteria included five specific appraisal questions unique to five study design categories where affirmative responses represent methodological quality (see Table 2). Thus, across the 27 studies, a total of 135 MMAT questions were coded. Of these, 121 were affirmative responses (89.6%) with 12 individual studies (44.4%) coded as affirmative for all five appraisal questions.

**Table 2:** MMAT appraisal

Quantitative descriptive studies					
**Article**	Question 1	Question 2	Question 3	Question 4	Question 5
	Is the sampling strategy relevant to address the research question?	Is the sample representative of the target population?	Are the measurements appropriate?	Is the risk of non-response bias low?	Is the statistical analysis appropriate to answer the research question?
Mosley-Johnson, Walker, 2022	Yes	Yes	Yes	Yes	Yes
Duk909e, 2021	Yes	Yes	Yes	Can't tell	Yes
Asgary, Beideck 2022	Yes	Yes	Yes	Yes	Yes
Massey, Zhong, 2023	Yes	Can’t tell	Yes	No	Yes
Liu, 2024	Yes	Yes	Yes	Yes	Yes
Hamilton, Patler 2022	Yes	Yes	Yes	Yes	Yes
Nikolaus, Cassandra et al., 2022	Yes	Yes	Yes	Can’t tell	Yes
Nikolaus, Hebert, 2022	Yes	Yes	Yes	Yes	Yes
Murillo, Reesor, 2017	Yes	Yes	Yes	No	Yes
Osborn, Albrecht, 2022	Yes	Yes	Yes	Can’t tell	Yes
Osborn, Morey, 2023	Yes	Yes	Yes	Yes	Yes
Weigel, Armijjos, 2018	Yes	Yes	Yes	Yes	Yes
Bermúdez-Millán, Wagner, 2019	Yes	Yes	Yes	Yes	Yes
Huang, Radha Saseendrakumar, 2023	Yes	Yes	Yes	No	Yes
Mullin, Brett, 2019	Can’t tell	Can’t tell	Yes	Yes	Yes
**Qualitative studies**					
**Article**	Question 1	Question 2	Question 3	Question 4	Question 5
	Is the qualitative approach appropriate to answer the research question?	Are the qualitative data collection methods adequate to address the research question?	Are the findings adequately derived from the data?	Is the interpretation of results sufficiently substantiated by data?	Is there coherence between qualitative data sources, collection, analysis and interpretation?
Rivers, Hingle, 2020	Yes	Yes	Yes	Yes	Yes
Miranda, Garcia, 2021	Yes	Yes	Yes	Yes	Yes
Joachim-Célestin, Gamboa-Maldonado, 2020	Yes	Yes	Yes	Yes	Yes
Brown, Perkison, 2018	Yes	Yes	Yes	Yes	Yes
Testerman & Chase. 2018	Yes	Yes	Yes	Yes	Yes
**Randomized controlled trials**					
**Article**	Question 1	Question 2	Question 3	Question 4	Question 5
	Is randomization appropriately performed?	Are the groups comparable at baseline?	Are there complete outcome data?	Are outcome assessors blinded to the intervention provided?	Did the participants adhere to the assigned intervention?
Ramirez & Wu, 2017	Yes	No	Yes	No	Can’t tell
Burner, Lam, 2018	Yes	Yes	No	No	Yes
**Quantitative, non-randomized**					
**Article**	Question 1	Question 2	Question 3	Question 4	Question 5
	Are the participants representative of the target population?	Are measurements appropriate regarding both the outcome and intervention (or exposure)?	Are there complete outcome data?	Are the confounders accounted for in the design and analysis?	During the study period, is the intervention administered (or exposure occurred) as intended?
Moyeda-Carabaza, Murimi, 2020	Can’t tell	Yes	No	Can’t tell	Yes
Oh, Ell, 2018	Yes	Yes	Yes	Yes	Yes
York, Kujan 2020	Can’t tell	Yes	Yes	No	Yes
Leining, Zhou, 2023	Yes	Yes	Yes	Yes	Yes
**Mixed-methods**					
**Article**	Question 1	Question 2	Question 3	Question 4	Question 5
	Is there an adequate rationale for using a mixed methods design to address the research question?	Are the different components of the study effectively integrated to answer the research question?	Are the outputs of the integration of qualitative and quantitative components adequately interpreted?	Are divergences and inconsistencies between quantitative and qualitative results adequately addressed?	Do the different components of the study adhere to the quality criteria of each tradition of the methods involved?
Page-Reeves, Shrum, 2019	Yes	No	No	No	Yes

*MMAT* Mixed Methods Appraisal Tool

### Sample and Setting

The aggregate number of participants across all studies was 7,528,452 (*n* = 596,982 were Hispanic/Latino). For articles that included multiple ethnicities (*n* = 10) [22–31], Latinos comprised, on average, 20.8% of the total sample (ranging from 6.2 to 80%). Of the articles including only Latino samples (*n* = 17), 7 articles reported specific Latino sub-populations, 5 of which described Mexican heritage, and only one study described multiple Latino subgroups (Mexican, El Salvadorian, Guatemalan) [32]. None of the studies that included multiple ethnicities described Latino subgroups by country of origin. Regions were predominantly general US (*n* = 8), Southwest (*n* = 7), and West Coast (*n* = 7) with 18 studies taking place in communities (e.g., national or state-wide surveys, churches, schools, community organizations) and the remaining studies taking place in outpatient clinical settings (*n* = 9). Twenty-six articles (96.3%) included only adult samples (age 18 +) with only one article (3.7%) focused on pediatric participants [25] and one (3.7%) focused solely on adults aged 65 and older [27].

### Social Needs

Social needs explored were food security (*n* = 16) [21, 25–27, 29–40], financial need (*n* = 8) [21, 22, 32, 41–43], transportation (*n* = 6) [22, 32, 34, 42–44], childcare (*n* = 5) [22, 32, 42–44], social support (*n* = 5) [24, 32, 38, 45, 46], housing (*n* = 2) [23, 28], legal (*n* = 2) [34, 47], and safety (*n* = 1) [32]. Most articles explored only one social need (*n* = 18) [23–28, 30, 31, 33, 35–37, 39–41, 45–47]; however, nine articles examined multiple social needs [21, 22, 32, 34, 38, 42–44].

#### Food Security

Articles describing the relationship between food insecurity and diabetes provided evidence to support food insecurity as a risk factor for T2D and prediabetes [26, 30, 31, 36, 37, 39]. All six studies measured food insecurity based off of the US household food security survey module developed by the US Department of Agriculture which is available in both English and Spanish [48]. Food insecurity experiences were described as causing emotional strain and as a barrier to both prevention and management of T2D [32, 34]. Interventions incorporating strategies to address food insecurity such as referrals to food banks suggest small reduction in HbA1c or food insecurity [21, 40].

##### Food Security and T2D Prevention and Risk

Four studies described food insecurity in the context of prediabetes and diabetes prevention [25, 29, 32, 34]. Duke et al. [25] found odds of prediabetes were greater in food-insecure Hispanic or Latino youth compared to their Non-Hispanic White food-insecure counterparts, Murillo et al.’s [29] study of low-income multi-ethnic adults found that prediabetes prevalence did not differ in Hispanic women or men by food security status. Food insecurity and food cost were identified as priorities to consider with diabetes prevention interventions in Brown et al.’s [34] qualitative study with Latinos. Consistent with these findings, Latinas involved in a culturally adapted DPP reported food insecurity as a barrier to adopting preventive behaviors, describing how healthy food was not affordable or available and would require redirecting critical financial resources [32].

Five studies linked food insecurity to increased T2D risk or prevalence when compared to food-secure counterparts [26, 30, 31, 36, 37, 39]. Food insecurity as associated with diabetes risk or prevalence persisted when comparing food-insecure Latinos (1) born in the US [36], (2) living in the US for more than 10 years [37], (3) when adjusting for age [26], and (4) when evaluating in Mexican immigrant adults [39].

The association between food insecurity and T2D risk was assessed in cross-sectional and longitudinal analyses using the same sample. In the cross-sectional analysis involving young adults (aged 24–32 years), food insecurity was associated with lower odds of developing T2D [31]. However, the longitudinal analysis showed that the presence of food insecurity during young adulthood was associated with increased T2D odds in middle adulthood [30].

Two studies described the nature of food insecurity and how it is experienced in the context of T2D by Latinos [35, 38]. Moyeda-Carabaza et al. [35] described food insecurity among Mexican-origin adults in a Texas border town participating in a Diabetes Empowerment Intervention. Of those originally from Mexico, 48.1% reported food insecurity and skipping or reducing meal sizes as coping strategies. Among participants originally from Texas, 21.1% reported food insecurity and eating less than they needed as a coping strategy [35]. Page-Reeves et al. [38] described the emotional toll of food insecurity among Latina immigrant women at risk for diabetes who reported experiencing “frustration, impotence, and sadness” in not being able to provide enough food for their children [38].

##### Food Security and T2D Management

Two articles described disparities experienced by Latinos managing T2D in the context of food insecurity. Rates of food insecurity in Hispanic Medicare beneficiaries with T2D were higher (20.1%) compared to the entire multi-ethnic sample (11.6%) [27]. Bermúdez-Millán et al. [33] found that Latinos with poorly controlled T2D (HbA1c > 8%) reporting food insecurity had significantly higher glucose, insulin, and insulin resistance than their food-secure counterparts. Further, food insecurity had significant direct effects on insulin resistance [33]. Two intervention studies incorporated strategies to address food insecurity among Hispanic/Latino individuals with T2D [21, 40]. York et al. [40] found that an organic vegetable medical prescription intervention improved food security in 12 of the 21 participants but did not result in a statistically significant improvement in HbA1c. Leining et al. [21] adapted a diabetes self-management education (DSME) intervention to include referrals to food banks and other resources for those screening positive for food insecurity. While HbA1c improved, food security status was not reevaluated in this study.

#### Financial Need

Articles exploring financial social needs (*n* = 7) [21, 22, 32, 34, 41–43] described finances as a barrier to diabetes prevention and management. For the purpose of this review, material need insecurity (MNI), defined as reduced ability to access or pay for basic needs [42], was categorized as financial need. One article evaluated the benefits of a diabetes self-management intervention incorporating linkages and resources for individuals with financial need [21]. Six of the seven articles discussing financial need included Latino-only samples and no articles analyzed the relationship between subjective financial need and diabetes prevalence or risk.

##### Financial Need and Diabetes Prevention

In Miranda et al.’s [41] study exploring the experiences of Mexican men at risk for T2D, participants described financial strain as a barrier to healthy behaviors where work and providing for the family was prioritized over personal health. Financial need was also identified as a priority consideration for diabetes prevention by Latinas and promotores participating in an adapted diabetes prevention program (DPP) [32] and Mexican Americans [34].

##### Financial Need and T2D Management

Mullin et al. [42] evaluated MNI in low-income Latino adults with T2D by asking, “During the last 12 months, have you spent less on food, heat or other basic needs so you would have enough money for your medicines?” A greater proportion of participants with MNI had poor glycemic control (40%) than those with material need security (MNS) (26%). Further, more patients with MNI reported medication non-adherence due to negative beliefs about medications and difficulty accessing care due to cost compared to those with MNS. Similarly, Latinos from Mexico and Central America with experience in DSME described money as a barrier to DSME participation [43]. However, in Huang et al.’s [22] study of multi-ethnic adults with diabetic retinopathy (13.2% Hispanic), delaying care due to cost was not significantly higher in Hispanic compared to Non-Hispanic White participants.

Leining et al. [21] evaluated the effects of a DSME program adapted to meet the needs of uninsured Hispanic adults. The program included referral to free clinics, resources for free to low-cost medications, and free lab work and consultations. Participants in the program experienced significant improvements in eye exam coverage; models suggested a projected decrease in HbA1c of 0.201% per month in the program.

#### Transportation

Six studies described transportation issues as they relate to diabetes prevention and management [22, 32, 34, 42–44]. Articles were predominantly qualitative (*n* = 4) and described perspectives on transportation as both a hypothetical and actual barrier to diabetes prevention and management. No articles evaluated subjective transportation needs in relation to diabetes prevalence or risk.

##### Transportation and Diabetes Prevention

When anticipating potential barriers to engagement in diabetes prevention, transportation was identified by both Mexican Americans [34] and a majority of Latina women participating in focus groups with a history of gestational diabetes [44]. Latina women participating in a modified DPP also identified unreliable transportation and the cost of gas as a barrier to their participation [32].

##### Transportation and T2DM Management

Mullin and colleagues [42] found that low-income Latinos with T2D reporting MNI had more difficulty accessing care due to a lack of transportation than those with MNS. Testerman and Chase’s [43] qualitative interviews with low-income Latino DSME participants reflected lack of transportation, inability to drive, or living far from the clinic as barriers to DSME participation. Huang et al.’s [22] evaluation of associations between transportation and diabetes outcomes in a multi-ethnic sample (13.2% Hispanic) did not identify disparities in transportation as causing delays in medical care between Hispanic and Non-Hispanic White participants with diabetic retinopathy. When comparing the odds of reporting transportation issues, results were not statistically significant between the two ethnic groups.

#### Childcare

Five studies discussed in prior sections evaluating food security and financial and/or transportation barriers also included discussion of childcare as a barrier to diabetes prevention and management [22, 32, 42–44]. None of the included articles examined childcare as the focus of the study design or evaluated associations between childcare needs and diabetes risk or prevalence.

##### Childcare and T2D Prevention and Prevalence

Lack of childcare was identified as a barrier to behavior modification in Latinas participating in the Vida Vibrante DPP [32]. In Rivers et al.’s [44] study soliciting feedback from Latinas about a hypothetical DPP program, 68% identified childcare as a potential barrier to participation.

##### Childcare and T2D Management

When evaluating multiple potential barriers to care in a multi-ethnic group of patients with diabetic retinopathy, the only statistically significant results reflecting social needs disparities in Hispanics indicated that Hispanic individuals had higher odds of delaying diabetes care due to childcare (OR = 8.04 [95% CI 2.26–31.9] *p* = 0.001) or elder care (OR = 3.78 [95% CI 1.10–12.0] *p* = 0.03) compared to Non-Hispanic Whites [22]. Further, Testerman and Chase’s [43] qualitative study found that lack of childcare during classes made participation in DSME more difficult for a sample of low-income Latinos. In Mullin et al.’s [42] study, low-income Latinos with T2D classified as MNI reported significantly higher rates of caregiving duties compared to those with MNS (OR 1.74 [1.16, 2.62]), suggesting a link between financial need and childcare or caregiving needs.

#### Social Support

Five articles evaluated social support in the context of diabetes prevention and management, three of which included a social support intervention [24, 32, 38, 45, 46]. While social support was described qualitatively as important for diabetes prevention and management, quantitative findings evaluating social support interventions were inconsistent.

##### Social Support and T2D Prevention

For Latinas participating in a DPP, “presence of strong social support” including family support was described as a factor in adoption of and adherence to preventive behaviors [32]. Page-Reeves et al. [38] evaluated the effects of a Social Isolation Support Group (SISG) in 21 Latina women (predominantly Mexican) on food security, HbA1c, perceived social support, and T2D risk. The number of respondents reporting having a person of trust in their lives increased but was not statistically significant; food security improved and was statistically significant. Qualitative findings described the SISG as helpful in forming social relationships, but changes in HbA1c or T2D risk were not reported.

##### Social Support and T2D Management

Ramirez and Wu [46] compared the effects of different phone messaging and social support interventions on physical activity and perceived social support in Latinos with T2D. Phone messaging interventions alone and those also incorporating family or friends’ social support demonstrated increases in physical activity from weeks 6 to 12. Burner et al. [24] found that a family support intervention in a predominantly Latino sample using text messaging increased days of self-monitoring of blood glucose by 1.6 days (compared to a decrease of 2 days in the control group). No statistically significant differences were found in tangible and emotional support or social connectedness outcomes between the intervention and control groups. However, qualitative findings described how patients in the intervention group reported making healthier decisions [24]. Data from a promotora-led T2D self-management support program found that changes in total social support were significantly correlated with self-efficacy (*p* = 0.01) in Hispanic patients with diabetes while social support was not significantly correlated with diabetes management behaviors [45].

#### Housing

Two studies identified associations of different housing needs with poorer T2D management [23, 28]. In a multi-ethnic sample using BRFSS data (7.1% Hispanic), Mosley-Johnson et al. [28] evaluated relationships between housing insecurity and diabetes processes of care and self-care behaviors. While Hispanic participants with housing insecurity did have a lower likelihood of having a physician visit in the past 12 months (0.49, 95% CI, 0.16-1.49) and a HbA1c check (0.35, 95% CI, 0.09-1.36), these were not significantly different when compared to Non-Hispanic White and Non-Hispanic Black participants. Asgary et al. [23] evaluated homelessness and diabetes management in a multi-ethnic sample (33.1% Latino). Among unhoused individuals, those with a HbA1c ≥ 8% were more likely to be Latino than their counterparts with a HbA1c < 8%.

#### Legal

Two studies evaluated legal status and diabetes outcomes in Latinos, both describing adults of Mexican descent and the influence of documented status and citizenship on diabetes prevention and prevalence [34, 47]. In Brown et al.’s [34] qualitative study exploring perceptions of diabetes, Mexican Americans with T2D identified fear of deportation as a priority issue to consider when developing interventions to prevent T2D. Hamilton et al. [47] evaluated health disparities in the immigration status of Latinx immigrant adults in California. Participants were classified as first-generation immigrants versus 1.5 generation (having arrived in the US at age 12 or younger), of Mexican descent or not, and as a citizen, Lawful Permanent Resident (LPR), or undocumented. When comparing groups within the 1.5 generation, predicted rates of diabetes in citizens were 10.5% compared to 15.1% in LPRs and 13% in undocumented individuals.

#### Safety

Safety was not studied alone by any of the included articles. Rather, the concept of safety as a social need surfaced in one qualitative study by Joachim-Célestin et al. [32] exploring factors associated with adoption and adherence to diabetes prevention behaviors in Latinas. In these focus groups, participants described a lack of safe and walkable areas as a barrier to engaging in physical activity.

## Discussion

The literature included in this integrative review supports the notion that certain social needs increase risk for diabetes (food insecurity, undocumented status) and negatively impact diabetes self-management (food insecurity, financial need, transportation, housing) in Latinos. Qualitative studies provided insights into the burdensome and complex experiences of specific social needs in Latinos at risk for or managing T2D. Clear gaps in the research suggest a need for studies comprehensively assessing social needs across the lifespan and implementing rigorous interventions that integrate social needs screening and referral processes. Few studies described person-centered demographics of Latinos that may influence social needs and/or access to related resources (e.g., citizenship, preferred language, Latino subgroup, acculturation, time in the US).

### T2D Prevention

Four of the five included qualitative studies explored how social needs influenced or could influence diabetes prevention interventions; however, more quantitative research analyzing associations between specific social needs and diabetes risk and related preventive behaviors is needed. Findings could aid in identifying priority areas for social needs referrals as they relate to T2D prevention interventions. Shame and stigma as they relate to social needs in diabetes prevention were briefly discussed in some of the included articles [32, 38]. These findings are consistent with prior research about perceptions of social needs assessments of individuals at federally qualified health centers (FQHCs) including reports of fear, mistrust, negative experiences reporting social needs but not receiving support, and misunderstanding of the purpose of social needs assessments [49]. These factors could directly influence the validity of social needs assessment as well as the efficacy and appropriateness of social needs resources.

All social needs discussed in this review were identified as important considerations for diabetes prevention programming, with transportation and financial needs as the most commonly identified social needs. These findings support prior research demonstrating use of screening and referral for unmet social needs as important in successful implementation of pediatric weight management interventions [50]. Of the six intervention studies included in this review, only the social support intervention presented by Page-Reeves et al. [38] described diabetes risk, but this was not presented as a diabetes prevention intervention. There is a clear need for more diabetes prevention interventions for Latinos that incorporate social needs assessment and referral. The data in this review do provide important considerations that could shape diabetes prevention interventions that screen and refer for unmet social needs; however, evaluation of interventions incorporating social needs screening and referral and the implementation process will be necessary to determine effectiveness of these programs.

### T2D Management

Findings from this review suggest that food insecurity, financial needs, transportation needs, and housing needs are associated with poorer T2D management. Social support, transportation, finances, and childcare were identified as important considerations for engagement in DSME, but only one qualitative study described barriers and facilitators of DSME engagement as they relate to social needs in Latinos [43]. Five of the six intervention studies included in this review were diabetes management interventions targeting food security, financial need, and social support [21, 24, 40, 45, 46]. T2D management interventions incorporating social needs screen and referral have been evaluated in broader populations including Latinos. Fitzpatrick et al. [51] evaluated the feasibility and acceptability of community health workers (CHWs) to connect minority and low-income adults with T2D to resources for reported social needs. When comparing navigation to social needs resources only to navigation and T2D self-management support, both groups had statistically significant improvements in HbA1c, and participants reported requesting more interaction with CHWs. Findings from this review and similar related research [52, 53] reinforce the importance of social care in concert with T2D care and the potential benefit of partnership with CHWs as social needs navigators and sources of social support [51, 54].

### Implications for Future Research

This integrative review supports the need for high-quality research about diabetes prevention and management among Latinos incorporating comprehensive social needs assessments and associated interventions. While a variety of social needs were explored in the included articles, certain social needs that were included in our search criteria such as employment and utilities insecurity were not clearly evaluated in any of the studies; safety as a social need was only briefly mentioned in relation to physical activity. Findings from Hamilton et al.’s [47] work suggests that documented status and length of time in the US could influence diabetes risk. However, research is needed to further understand intersections of other social needs with documented status and whether this influences access to resources to address social needs. Additionally, while documented status is a main issue of concern impacting Latinos, it is not the only legal issue Latinos face [55]. More research evaluating the association of broader legal needs with diabetes prevention and management in Latinos could further inform tailored diabetes interventions. None of the included articles utilized a complete social needs assessment such as PRAPARE [56] or Your Current Life Situation [57] which screen for social needs such as housing, food security, employment, and financial need. Research that more clearly evaluates all social needs as well as the nature of less commonly studied social needs in Latinos is needed to advance this science. Additionally, a deeper understanding of social need clusters, priorities, factors that moderate and mediate the influence of social needs on diabetes outcomes is needed [5]. Some scholars propose moving from screen and refer approaches to direct social need intervention by providers [58] and designing healthcare delivery to incorporate social care [52].

This review also sheds light on the importance of outcome measures in diabetes and the evaluation of social needs. While most intervention studies included in this review measured HbA1c, few results reached significance. Evaluation of social needs outcomes (reevaluation of social needs, intervention uptake, perceptions of the social need intervention) was much less common and would provide meaningful insight into potential intervention adaptations. While this review suggests a need for more research incorporating comprehensive screen and referral for social needs in diabetes management interventions, designs must consider the complexity of social needs screening. Prior successful efforts to systematically screen and refer for social needs in people with cystic fibrosis involved coordinated efforts to generate reminders, offered multiple screening formats, and utilized a tailored disease-specific screening instrument.

A majority of studies included in this review did not identify Latino subgroups or other important demographic data beyond Latino ethnicity such as years in the US, preferred language, or acculturation. Person-centered research methodologies informed by intersectionality are especially important in the social needs space considering that root causes of social needs are often shaped by multiple co-occurring factors. Sociocultural protection, documentation status, education, and ethnic identity have been found to vary significantly between Latino subgroups and may predict health risk behaviors [59].

Strikingly, only one of the included articles used the term, “social needs,” and none of the articles provided a definition of the term. To advance social needs science as it relates to diabetes in Latinos, scholars must continually reference and reevaluate social needs as a construct. Social needs are experienced by individuals in a societal context—thus, they will change over time as societal norms and conditions evolve. For example, some suggest internet access should be considered a social need [60]—a concept that arguably would not have been deemed a priority when the DPP and DSME were first developed. Today, internet access provides individuals with telemedicine resources, thus making remote diabetes prevention and self-management education possible [61]. When the social need of the internet is met and individuals can access care and resources virtually (e.g., telehealth, virtual programs), the burden of other social needs may be lessened [62]. Thus, as diabetes prevention and management efforts in Latinos advance, methodologically rigorous incorporation of social needs assessment and interventions must also evolve.

### Strengths and Limitations

This is the first review synthesizing the science of social needs as they relate to diabetes in Latinos. Our rigorous search strategy and review process produced important evidence to inform understanding of diabetes disparities among Latino populations. This review is not without limitations. The search strategy, although reviewed by a health sciences librarian, may have not elicited all relevant research. Further, articles that did not disaggregate findings to Latinos were excluded. Research conducted outside the US in Latino populations may have offered more perspective on social needs screening and referral.

## Conclusion

This review provides important insight into the influence of social needs on T2D in Latinos. Diabetes disparities science must advance in concert with social needs science to inform policy and public health efforts. Diabetes prevention and management efforts for Latinos must effectively assess social needs and intervene using appropriate, sustainable and effective strategies to ultimately eliminate diabetes disparities.

## Data Availability

All data used in the review process, such as full data extraction tables, are available from the corresponding author upon request.
